# Case Report: Chemotherapy, immunotherapy, and radiotherapy achieve complete response for metastatic ureteral urothelial carcinoma with significant squamous differentiation

**DOI:** 10.3389/fonc.2026.1702896

**Published:** 2026-02-02

**Authors:** Ying Bi, Jun Li, ShangHu Qu, Jun Yin, Yong Zhu, ChongJian Zhang, Yu Bai

**Affiliations:** Department of Urology, Peking University Cancer Hospital Yunnan&Yunnan Cancer Hospital&The Third Affiliated Hospital of Kunming Medical University, Yunnan, China

**Keywords:** chemotherapy, combination therapy, immunotherapy, radiotherapy, significantly squamous differentiated urothelial carcinoma

## Abstract

**Background:**

The majority of tumors in the upper urinary tract are classified as urothelial carcinomas, with only 15% exhibiting different levels of squamous differentiation, known as the squamous subtype. These tumors tend to be more aggressive and are associated with a worse prognosis compared to standard urothelial carcinomas.

**Case presentation:**

A 60-year-old man was found to have cancer in his left ureter and subsequently underwent a robot-assisted laparoscopic procedure to partially remove the left ureter and perform a ureterovesical anastomosis. The postoperative analysis revealed high-grade urothelial carcinoma with marked squamous differentiation(40% squamous differentiation) and microsatellite stability (MSS). Three months after surgery, the abdominal incision recurred and abdominal mass resection was performed again. One month after abdominal surgery, imaging evaluation showed lymph node metastasis in the left lower wall of the bladder and retroperitoneum.Ultimately, the patient attained complete imaging remission following treatment with the \GC\ chemotherapy regimen, along with \tislelizumab\ immunotherapy and radiotherapy.

**Conclusion:**

The \GC\ chemotherapy protocol, along with \tislelizumab\ immunotherapy and radiotherapy, shows remarkable effectiveness in treating metastatic ureteral urothelial carcinoma with significant squamous differentiation.

## Background

Upper tract urothelial carcinoma (UTUC) is a highly uncommon condition. In Europe and the United States, its occurrence is roughly 1–2 cases per 100,000 individuals. UTUC represents about 5-10% of all urothelial carcinomas. Among these, urothelial carcinoma exhibiting varying levels of squamous differentiation, known as the squamous subtype, constitutes around 15% of the total (1). This squamous differentiation is closely linked to several negative prognostic indicators, such as tumors graded at histological grade 3, the presence of lymphovascular invasion, concurrent carcinoma *in situ*, advanced tumor stages, and the development of lymph node metastases (2). For patients with locally high-risk UTUC, the primary treatment options include radical nephroureterectomy (RNU) and excision of the bladder cuff. Nevertheless, the rate of local recurrence post-surgery is notably high, with late recurrences potentially reaching 30%. Particularly in T3 and T4 classifications, patients with high-grade tumors and multiple recurrences face an elevated risk of recurrence, and those who experience local recurrences frequently encounter metastatic recurrences, resulting in poor overall survival rates (3).

Currently, chemotherapy using platinum compounds is the primary approach for treating advanced or metastatic urothelial carcinoma. For those patients who can handle cisplatin, a combination of cisplatin and gemcitabine is advised. In recent years, however, the FDA has authorized several immune checkpoint inhibitors (ICIs) for managing urothelial cancers originating from the bladder or other parts of the urinary system. Among the ICIs approved for urothelial cancer are drugs targeting programmed cell death protein-1 (PD-1), such as nivolumab and pembrolizumab, as well as those targeting programmed cell death ligand 1 (PD-L1), including atezolizumab, avelumab, and durvalumab (4). Tislelizumab, a monoclonal antibody against PD-1, was assessed in a single-arm, phase 2 trial (NCT04004221/CTR20170071) for its safety, tolerability, and effectiveness in patients with PD-L1-positive urothelial carcinoma. The findings indicated that tislelizumab provided notable clinical advantages for patients who had previously undergone treatment for locally advanced or metastatic PD-L1-positive urothelial carcinoma, with manageable safety profiles (5). Nonetheless, there is still a scarcity of research regarding the use of radiotherapy for upper tract urothelial carcinoma (UTUC), leaving the effectiveness and safety of palliative, salvage, and adjuvant radiotherapy uncertain.

This report discusses a case involving recurrent metastatic ureteral urothelial carcinoma characterized by notable squamous differentiation following surgical intervention. The patient underwent a combination of GC chemotherapy, tislelizumab immunotherapy, and radiotherapy. This particular treatment approach has not been documented previously for metastatic ureteral urothelial carcinoma with marked squamous differentiation. Remarkably, the patient attained a clinical complete remission (CR), with a progression-free survival (PFS) lasting an impressive 46 months, greatly prolonging the survival duration for similar cases.

## Case presentation

**Figure 1:**
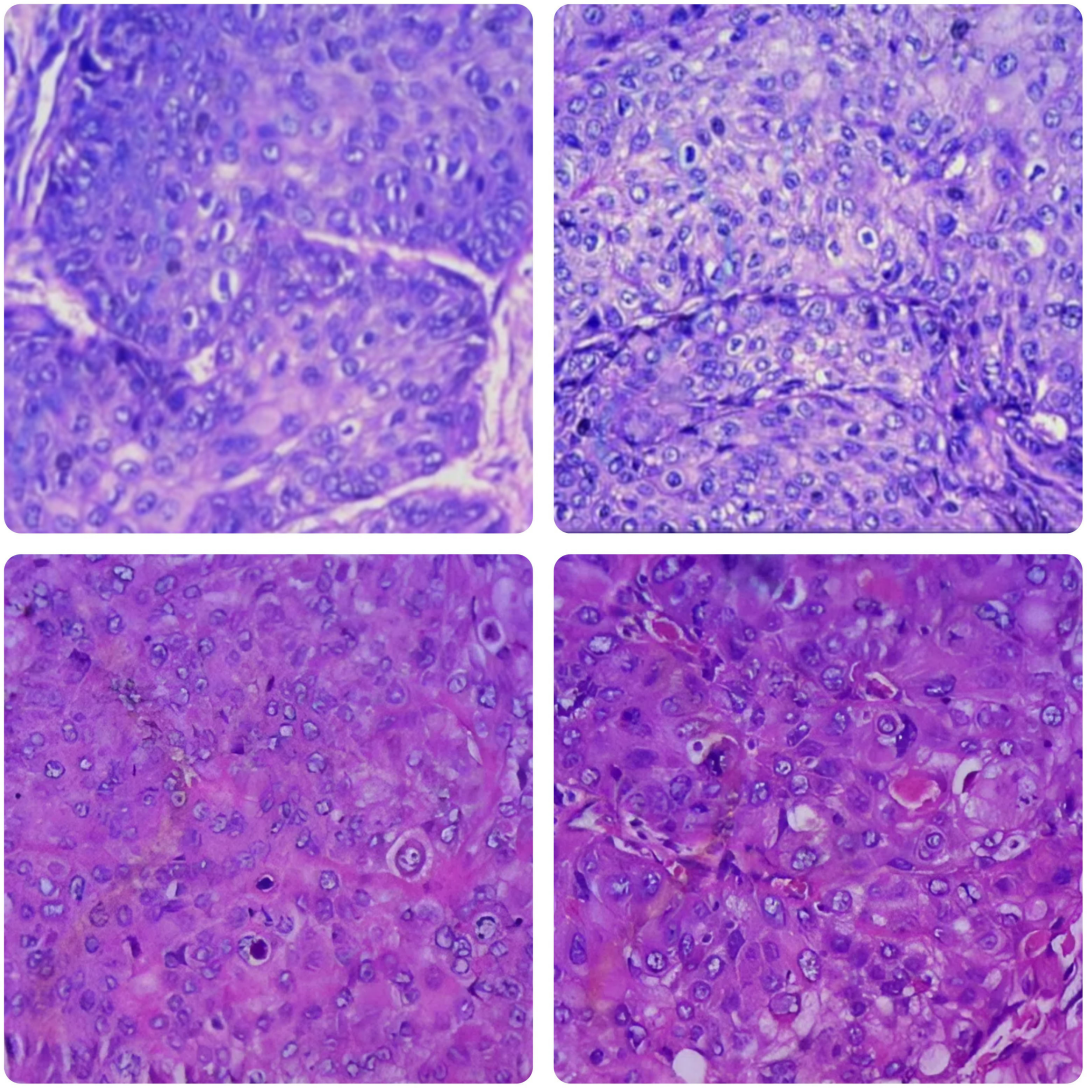
Microscopic revealing urothelial carcinoma with significant squamous differentiation (H&E stain, ×100).

**Figure 2:**
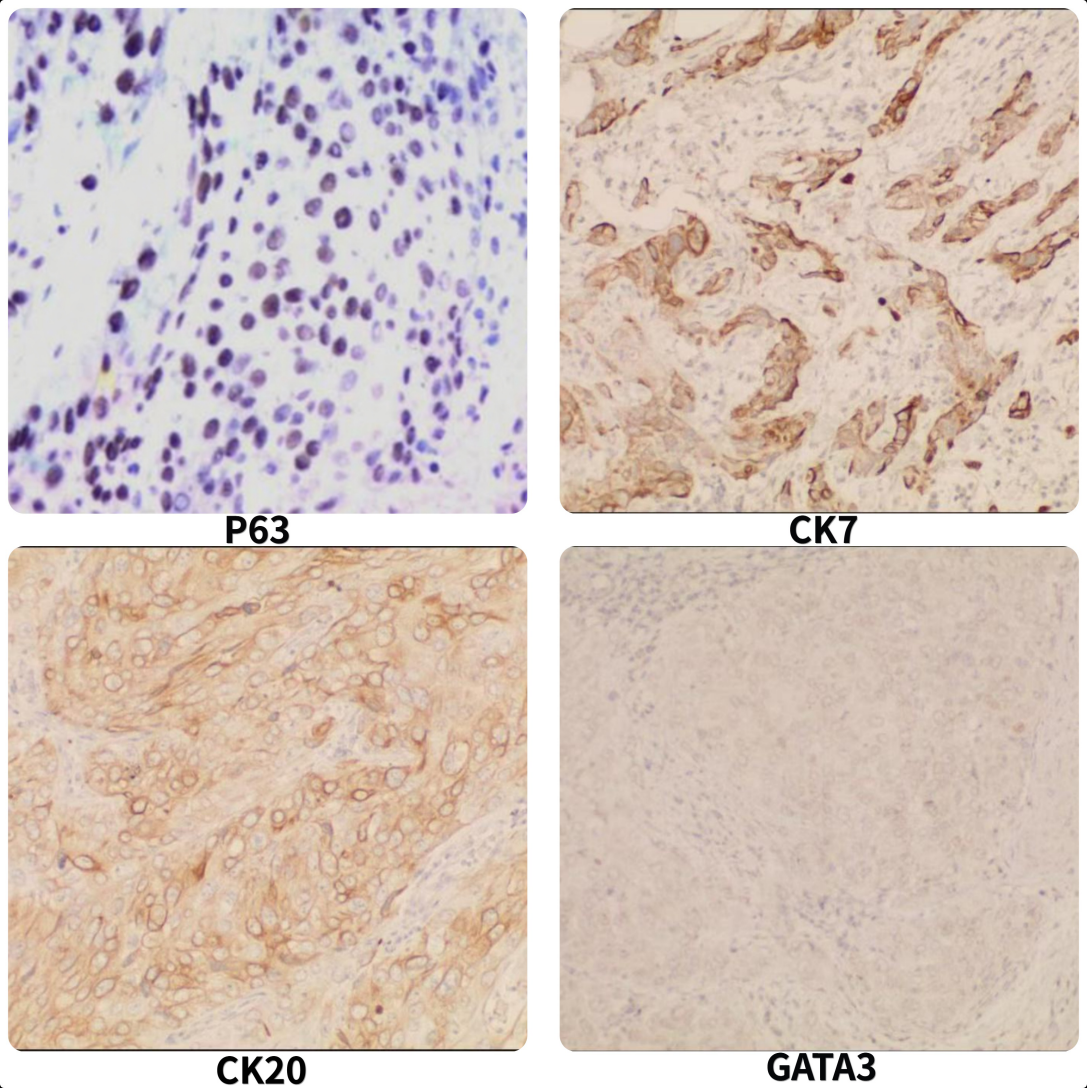
Microscopic revealing p63(+), CK7(+), CK20 (+), GATA3 (focal +); (IHC stain, ×100).

**Figure 3:**
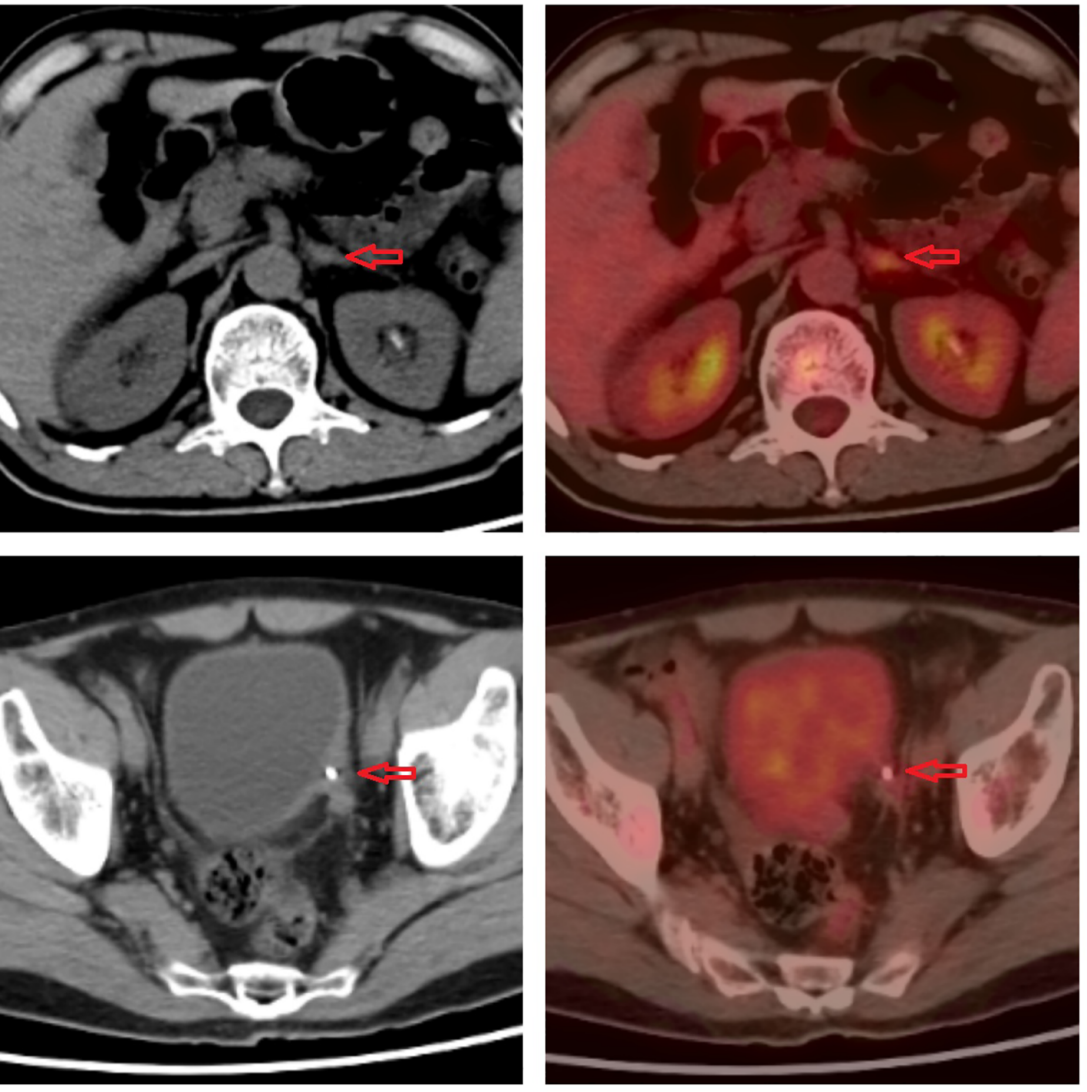
Imaging of 18F-fluorodeoxyglucose positron emission tomography.

**Figure 4:**
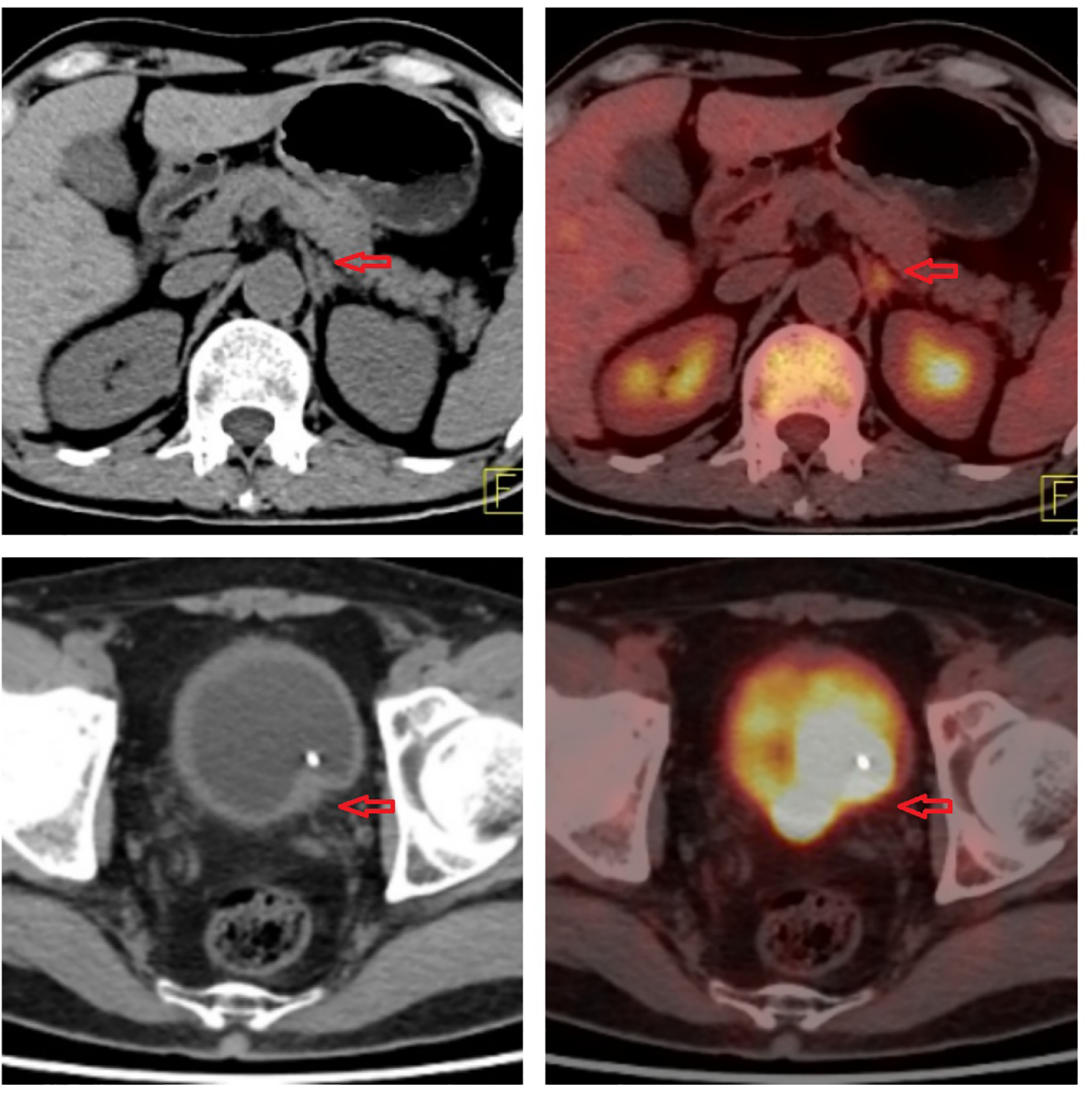
Imaging of 18F-fluorodeoxyglucose positron emission tomography.

**Figure 5:**
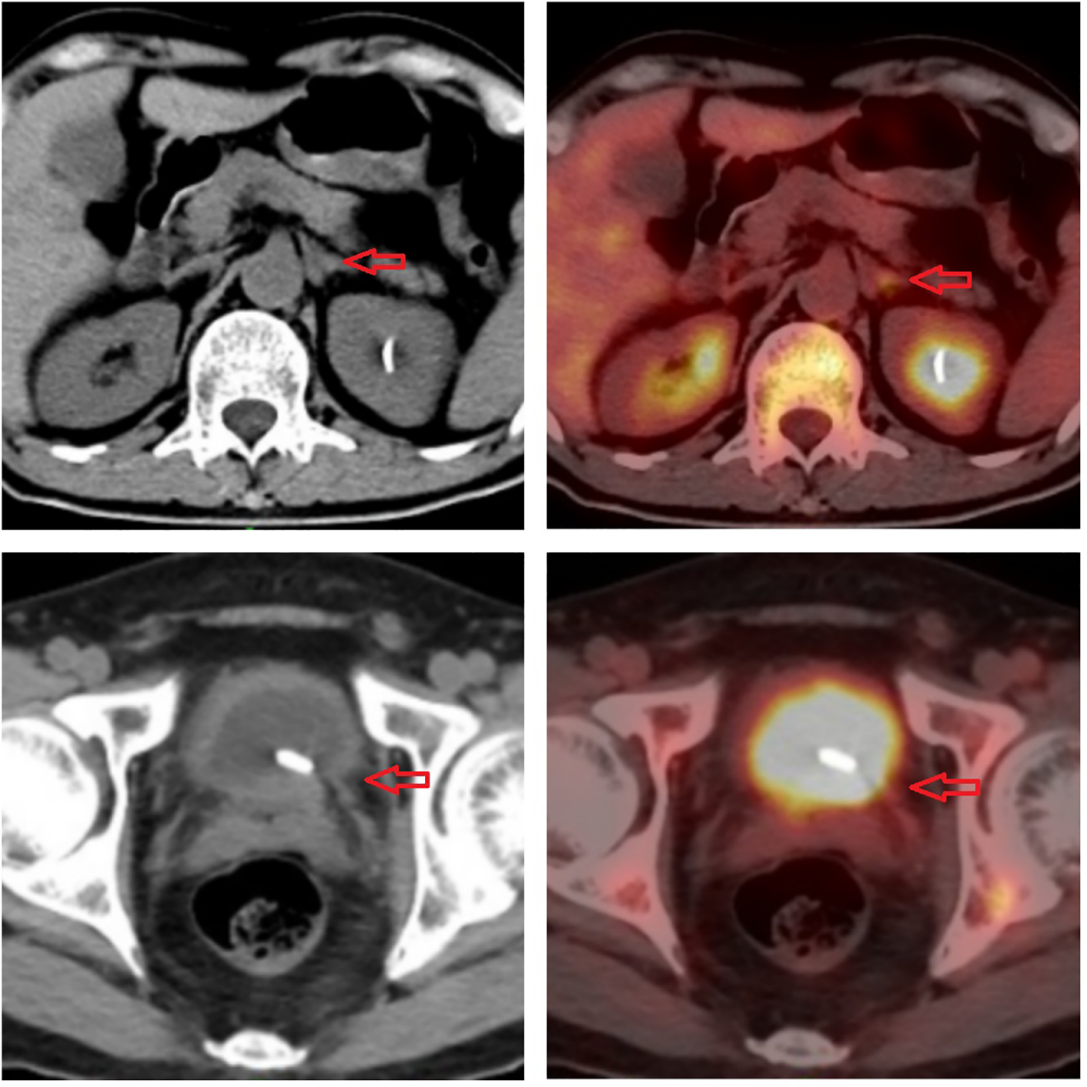
Imaging of 18F-fluorodeoxyglucose positron emission tomography.

**Figure 6:**
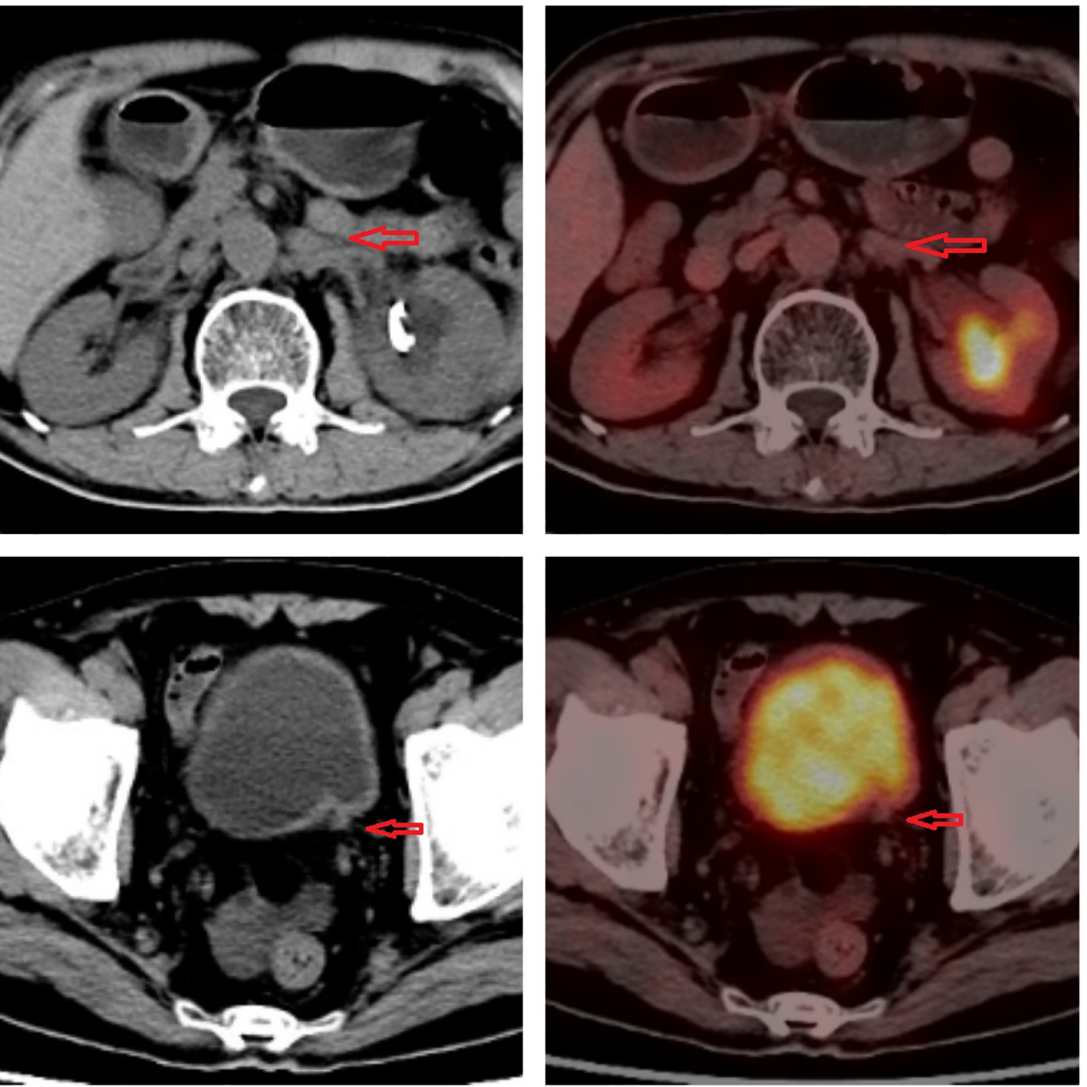
Imaging of 18F-fluorodeoxyglucose positron emission tomography.

**Figure 7:**
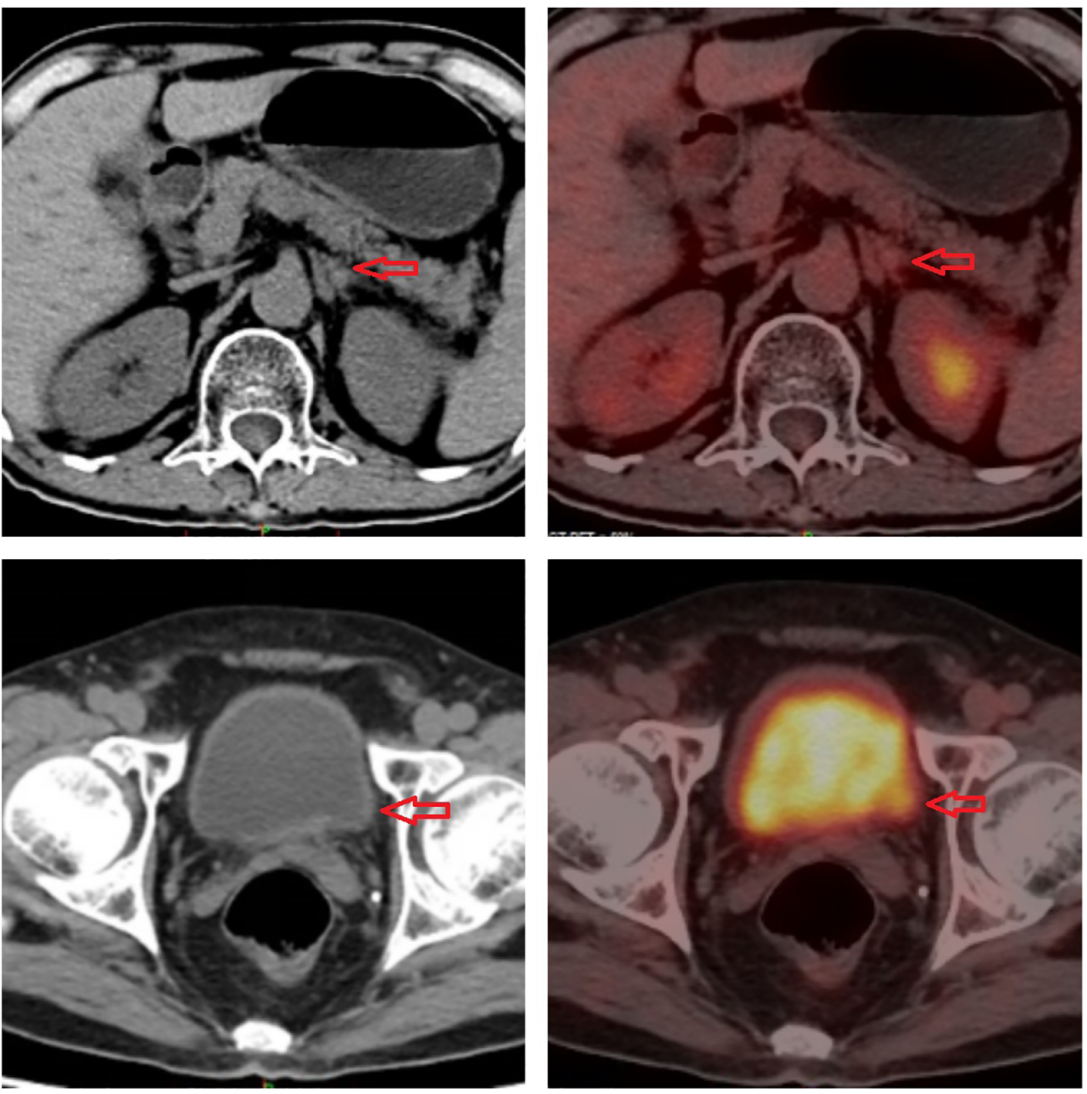
Imaging of 18F-fluorodeoxyglucose positron emission tomography.

In January 2021, a 60-year-old man sought treatment at a tertiary facility in Kunming, Yunnan Province, presenting with gross hematuria for 2 weeks. He had no notable medical history, including no family history of diseases, smoking, or occupational hazards. A CT scan revealed a tumor and blockage in the lower part of the left ureter, measuring around 2*1 cm, along with hydronephrosis in the left kidney. Urine cytology indicated the presence of cancer cells, leading to a diagnosis of left ureteral cancer. On January 29, 2021, he underwent robot-assisted laparoscopic partial resection of the left ureter and ureterovesical anastomosis under general anesthesia. The postoperative pathology report identified high-grade urothelial carcinoma with significant squamous differentiation (40% squamous differentiation), microsatellite stability(MSS),PD-L1(CPS:3),CK5/6(+),CK20(+),GATA3(focal+),P53(-),CK7(+),p63(+), (**Figures 1**, **2**); Chronic inflammatory infiltration of the mucosa surrounding the tumor. The tumor had invaded the entire ureteral wall and involved nearby adipose tissue, but there was no cancer at the specimen’s ends or evidence of lymphatic or vascular invasion, classified as pathological stage pT3. Following surgery, the patient received nine intravesical BCG vaccine instillations. On April 14, 2021, three months post-surgery, a mass was detected above the umbilicus, prompting another hospital visit. Ultrasound revealed a solid structure in the subcutaneous fat layer, possibly an inflammatory granuloma, but metastatic disease could not be ruled out. A CT scan showed a mass in the lower left ureter, hydronephrosis, and a nodular lesion in the abdominal wall above the umbilicus, raising concerns about cancer recurrence. On April 23, 2021, he underwent cystoscopy under general anesthesia, ureteroscopy, transurethral ureteral stent placement, and abdominal mass resection. The pathology report confirmed a malignant tumor consistent with high-grade urothelial carcinoma with significant squamous differentiation. The patient, anxious about his health, visited our hospital on May 6, 2021.A PET/CT scan indicated thickening of the left ureter’s soft tissue and the adjacent bladder wall, with increased metabolic activity suggesting possible malignancy. Multiple retroperitoneal lymph nodes showed increased metabolism, indicating potential metastasis (**Figure 3**). The diagnosis was left ureteral cancer with retroperitoneal lymph node metastasis, leading to a treatment regimen of tislelizumab 200mg and a GC chemotherapy protocol (gemcitabine 1g d1,d8;platinum 50mg d1-2) for five cycles. On August 30, 2021, after the treatment, a follow-up PET/CT showed no significant metabolic increase in the left ureter or bladder wall, some retroperitoneal lymph nodes remained unchanged, with some showing increased metabolism (**Figure 4**). The patient’s condition had slightly improved. However, on September 11, 2021, he experienced severe bone marrow suppression from chemotherapy, leading to its chemotherapy cessation while continuing immunotherapy. A new treatment plan was initiated, and on October 18, 2021, he received radiotherapy for the retroperitoneal lymph nodes, using the TOMO technique with a total planned dose of 48Gy over 30 fractions(Total radiation dose and fractionation mode: DT 95%PTV: 48Gy/1.6 Gy/30f, 95% PGT-N: 60Gy/2Gy/30f). After 20 sessions, he developed grade IV bone marrow suppression, resulting in a pause in radiotherapy. The actual doses delivered were lower than planned(actual total radiation dose and fractionation mode:95%PTV: 32Gy/1.6Gy/20f, 95%PGT-N: 40Gy/2Gy/20f), and during this period, he also received small doses of chemotherapy(cisplatin 49.653mg d1) and immunotherapy. On December 1, 2021, a PET/CT re-evaluation showed slight thickening of the left ureter walls with no significant metabolic increase, likely inflammatory. Some small retroperitoneal lymph nodes showed reduced size and metabolism, indicating a partial response to treatment(**Figure 5**). The patient’s condition improved significantly, achieving complete remission on imaging for the first time. Following the completion of radiotherapy, he continued with immunotherapy. On March 1, 2022, another PET/CT scan indicated a slight reduction in the left ureter wall thickening and no abnormal metabolic activity, suggesting inflammation. The retroperitoneal lymph nodes were slightly smaller, with no increased metabolism, indicating further inhibition of metastatic activity (**Figure 6**). The patient continued immunotherapy.By April 21, 2023, a follow-up PET/CT showed slight thickening of the upper left ureter wall without significant metabolic increase, indicating inflammation, and no enlarged lymph nodes were detected (**Figure 7**). Imaging suggested complete remission. The patient has since been regularly monitored at an external facility, with the latest follow-up on March 12, 2025, showing no signs of tumor recurrence or metastasis. He currently enjoys good renal function, a high quality of life, and is able to participate in moderate physical activity, with improved bone marrow function.

## Discussion

Ureteral cancer exhibits a significant level of aggressiveness. The ureter is surrounded by numerous lymphatic vessels, and its wall is relatively thin. This allows tumors to readily invade the muscle layer, leading to local spread and the potential for distant metastasis. As a result, ureteral cancer frequently recurs and metastasizes. The outcome for patients is often closely linked to the tumor’s grade and stage (6).

Squamous metaplasia is an adaptive change in the urinary tract epithelium that occurs under chronic stimuli such as inflammation, stones, and chemotherapy drugs, characterized by the replacement of the urinary tract epithelium with squamous epithelium. When metaplastic epithelium further accumulates gene mutations, it may progress to urothelial carcinoma with squamous differentiation. Therefore, metaplastic epithelium can be regarded as the “soil” for carcinogenesis. Urothelial carcinoma exhibiting squamous differentiation is characterized by a specific area of squamous differentiation within the typical urothelial carcinoma framework. This differentiation can present as keratinization, which includes the formation of keratinized beads, or through the existence of intracellular bridges. When a tumor consists solely of squamous cells without any urothelial elements, it is classified as pure squamous cell carcinoma. This type of urothelial carcinoma with squamous differentiation falls under the category of Variant Histology (VH) and is considered a high-risk feature, linked to increased chances of recurrence, progression, and poorer survival rates (7). Consequently, urothelial carcinoma with squamous differentiation is distinct from squamous cell carcinoma in terms of risk factors, molecular traits, and treatment approaches. Additionally, community pathologists have a significant missed diagnosis rate of 44% for VH in urothelial carcinoma (8), highlighting the importance of careful diagnosis.

Research analyzing RNA expression in the urothelial and squamous areas of identical tumors found that around 25% of the instances showed distinct molecular subtypes in these regions (9). This highlights significant intratumoral diversity, complicating the development of effective treatment approaches.

Currently, numerous studies have established that various anti-cancer therapies, including chemotherapy, immunotherapy, and targeted treatments, demonstrate effective results in cases of uncomplicated urothelial carcinoma. Nonetheless, the majority of existing research tends to concentrate on lower urinary tract urothelial carcinoma and uncomplicated forms, leaving upper urinary tract urothelial carcinoma and those with pronounced squamous differentiation underexplored. Tumors in the upper urinary tract are infrequent and have a low occurrence rate. Urothelial carcinoma exhibiting significant squamous differentiation is closely linked to several negative prognostic indicators (10). Research has shown that this type of carcinoma tends to have a worse response to treatment and a heightened likelihood of disease advancement compared to pure urothelial carcinoma. Currently, the most effective approach for managing upper urinary tract tumors is early detection followed by radical surgical intervention, as the presence of distant metastasis often correlates with unfavorable treatment outcomes. In this case, the patient presented with a rare case of metastatic ureteral urothelial carcinoma featuring significant squamous differentiation. Following a combination of chemotherapy, immunotherapy, and radiotherapy, the patient achieved complete remission.

In cases of ureteral cancer, surgeries aimed at preserving the kidney are generally discouraged. This is partly due to the often unclear extent of tumor infiltration prior to surgery, which can lead to an underestimation of the cancer’s stage. Additionally, whether performed through open techniques or endoscopy, merely excising the tumor to save the kidney can result in a recurrence or metastasis rate ranging from 15% to 80% in patients post-surgery. Consequently, kidney-sparing procedures are strictly indicated only for tumors situated in the collecting system of a solitary kidney, bilateral upper urinary tract epithelial tumors, and instances of severely compromised renal function (11). In this case, postoperative pathology revealed that the tumor had invaded all layers, indicating a locally advanced stage. Thus, retaining the kidney solely because the tumor was limited to the ureter was not justified. This led to the patient experiencing a swift recurrence and retroperitoneal lymph node metastasis within three months following the surgery. Since the patient initially sought care elsewhere and had already developed distant metastasis by the time they arrived at our facility, only palliative treatment options were available. Given the patient’s condition and existing guidelines, a regimen of GC chemotherapy along with tislelizumab immunotherapy was initiated. After five cycles of this platinum-based chemotherapy combined with immunotherapy, the patient could not continue due to severe bone marrow suppression, a common side effect of GC regimens. Tolerance to chemotherapy varies among individuals, and the intensity of bone marrow suppression can differ based on a patient’s health status, chemotherapy dosage, and treatment frequency. Bone marrow suppression is categorized from grades 0 to 4, with severe cases potentially leading to fatal outcomes. Chemotherapy agents can impair the function or induce apoptosis in hematopoietic stem and progenitor cells, disrupting the bone marrow’s microenvironment (12). Inadequate management of bone marrow suppression during chemotherapy can hinder further treatment, delay progress, and prevent timely interventions. For patients experiencing grade 1–2 suppression, adjustments in chemotherapy dosages may help. However, for those with grade 3 or 4 suppression, physicians must evaluate the feasibility of continuing the current chemotherapy plan, as it could result in irreversible consequences, including death. At that point, the patient had reached grade IV bone marrow suppression, leading to the conclusion that continuation of the GC regimen was unviable. Research indicates that incorporating tislelizumab into the GC regimen significantly raises the risk of thrombocytopenia (13), prompting the decision to pursue adjuvant radiotherapy instead.

In the management of advanced ureteral cancer, the current recommendation is to utilize systemic chemotherapy that incorporates platinum-based agents. Following 4 to 6 cycles of gemcitabine paired with cisplatin or carboplatin, if the disease remains stable, avilumab immunotherapy may be continued. For patients who cannot tolerate platinum-based treatments and are PD-L1 positive, pembrolizumab or atezolizumab is advised. Currently, there is limited clinical data supporting the efficacy of adjuvant radiotherapy (14). Additionally, research indicates that among the various forms of urothelial carcinoma, tumors with squamous differentiation exhibit elevated PD-L1 expression in their cells, suggesting these tumors might respond more favorably to immune checkpoint inhibitors (15). Some studies also indicate that the presence of squamous or glandular in bladder urothelial carcinoma does not confer resistance to neoadjuvant chemotherapy with MVAC. In fact, patients with such mixed tumors may experience greater survival advantages from neoadjuvant chemotherapy compared to those with pure urothelial carcinoma. Thus, mixed histological characteristics should not be a deterrent to neoadjuvant chemotherapy; rather, they could serve as an indication for it (16). Numerous recent studies have shown that combining immunotherapy with chemotherapy offers several benefits. This approach can counteract the immunosuppression associated with advanced cancers, enhance the cross-presentation of tumor antigens, stimulate the growth of cytotoxic T cells, and increase their ability to eliminate tumor cells, while also mitigating some of the chemotherapy’s toxic effects and reducing the likelihood of drug resistance. Furthermore, the combination of immunotherapy and chemotherapy has yielded superior outcomes compared to monotherapy in treating various cancers (17). However, the upregulation of mannose-6-phosphate receptors (MPR) on tumor cell surfaces due to chemotherapy can activate cytotoxic T lymphocytes, allowing Granzyme B (GrzB) from CTLs to penetrate tumor cells more effectively, although this upregulation is transient. Therefore, timing is crucial when integrating chemotherapy with immunotherapy (18). Approximately 50% of patients with upper tract urothelial carcinoma (UTUC) have compromised renal function, which restricts the use of cisplatin. Consequently, some studies have indicated that tislelizumab, either alone or in combination with antibody-drug conjugates (ADCs), shows therapeutic promise for UTUC patients with renal impairment. Additionally, the combination of tislelizumab with or without gemcitabine-cisplatin (GC) chemotherapy regimens has demonstrated significant clinical benefits and manageable safety profiles for high-risk UTUC patients (19–21).

Currently, advancements in radiological technology have enabled the implementation of precise targeted and intensity-modulated radiotherapy (IMRT) for tumors, which safeguards vital normal tissues while delivering high doses to tumors, thus enhancing tumor eradication. A retrospective analysis involving 174 patients with recurrent or metastatic upper tract urothelial carcinoma (UTUC) who underwent salvage and palliative radiotherapy revealed a response rate of 68.8%, demonstrating the feasibility and effectiveness of this treatment approach. Patients who experienced remission through IMRT may benefit from improved survival rates with salvage and palliative radiotherapy. The presence of PD-L1 may influence radiosensitivity. Combining high-dose radiotherapy with concurrent chemotherapy could enhance treatment responses (22). Research indicates that radiotherapy not only induces tumor cell death but also stimulates innate immune cell activity, effectively activating cytotoxic T lymphocytes (CTL) and producing distant effects that can eliminate some metastatic tumor cells (23). Additionally, boosting immune function via immunotherapy may further improve radiotherapy outcomes and increase the likelihood of distant effects. Nonetheless, additional studies are required to explore the optimal sequencing of radiotherapy with immunotherapy and the appropriate fractionation of radiotherapy doses.

In this case, the patient could not endure chemotherapy due to significant bone marrow suppression following five cycles of platinum-based chemotherapy paired with immunotherapy. Bone marrow suppression is a fairly frequent side effect associated with GC chemotherapy protocols. Nonetheless, the intensity of this suppression can differ based on the patient’s overall health, the selected chemotherapy dosage, and the total number of treatment cycles. At that point, the patient had reached grade IV bone marrow suppression, making it impossible to proceed with the GC chemotherapy regimen, leading to the decision to implement adjuvant radiotherapy. Throughout this time, immunotherapy was consistently administered. The most recent follow-up revealed complete remission on imaging, reinforcing the idea that there is a synergistic relationship between chemotherapy and immunotherapy, as well as between immunotherapy and radiotherapy, which collectively produce a therapeutic effect that surpasses what could be achieved through any single modality. Additionally, notable advancements have been made in the management of advanced ureteral cancer, contributing valuable treatment strategies and clinical insights for this condition.

There is a consensus set of six molecular classes: luminal papillary (24%), luminal nonspecified (8%), luminal unstable (15%), stroma-rich (15%), basal/squamous (35%), and neuroendocrine-like (3%) (24). Recent evidence suggests that molecular classification of UC may provide more precise stratification that could impact treatment and patient survival. Because of the differences in the biological properties of UC subtypes, it is assumed that subtypes will exhibit differences in sensitivity or resistance to therapy. We performed partial molecular marker assessments on the patient, revealing results of PD-L1 (CPS: 3), CK5/6(+), CK20(+), GATA3 (focal +), and P53 (-). The majority of these markers are associated with luminal and basal molecular classifications. The research examined mRNA in muscle-invasive bladder cancer (MIBC) and confirmed through immunohistochemistry (IHC) the presence of a double-negative subtype, marked by low levels of both luminal and basal molecular markers, which correlates with a poor prognosis (25). Mutant p53 is linked to resistance to chemotherapy and is recognized as a negative prognostic indicator in bladder cancer (26). Based on the expression of biomarkers in the patient’s molecular classification, it can be deduced that the prognosis is relatively favorable, aligning with the positive treatment outcomes observed post-therapy.

When ureteral cancer penetrates the muscle layer, the outlook for patients tends to be unfavorable. The five-year survival rates vary significantly: 86% for T1NO, 77% for T2NO, 63% for T3NO, and a mere 39% for T4NO/Tx N1-3 (27). Research by Koie T and colleagues, utilizing a comprehensive national database from the Japanese Urological Association that included 348 medical institutions, revealed that individuals with upper urinary tract urothelial carcinoma at clinical stage T3 have a notably poorer prognosis compared to those at stage T2 (28). Implementing extensive lymph node dissection in a meticulous anatomical manner during surgery for this type of cancer can enhance staging accuracy, as lymph node involvement serves as an independent factor predicting lower survival rates (29). Bolenz C and associates discovered that a lymph node density of 30% or more correlates with increased five-year recurrence rates (25% vs. 38%; P = 0.021) and elevated cancer-specific mortality rates (30% vs. 48%; P = 0.032) (30). Additionally, Mason RJ and his team found that the ratio of positive to resected lymph nodes significantly impacts disease-specific survival (DSS) (HR: 2.70; P = 0.001), recurrence-free survival (RFS) (HR: 1.94; P = 0.015), and overall survival (OS) (HR: 2.34; P = 0.013), with a notable decrease in correlation when the cutoff is below 20%, leading to significantly lower DSS, RFS, and OS in N+ patients (31). Duquesne I posits that the benefit of lymph node dissection for N0 and Nx patients remains uncertain, suggesting that pN+ patients do not gain advantages from such procedures. The total count of lymph nodes removed during dissection is not an effective standalone prognostic indicator; rather, lymph node density proves to be a more precise measure for predicting outcomes post-dissection (32). In this case, the patient experienced recurrence and retroperitoneal lymph node metastasis, leading to a preference for systemic treatment over lymph node dissection. Although the patient achieved complete remission through a combination of three treatment modalities, the follow-up duration is relatively brief, presenting a limitation. Ongoing monitoring of the patient’s condition is essential, and further multi-center, large-scale studies are necessary to assess the effectiveness and safety of this combined treatment approach.

## Conclusion

A patient with advanced squamous differentiated metastatic ureteral urothelial carcinoma, which recurrence following a kidney-sparing procedure, was managed at our facility. The treatment involved a salvage chemotherapy regimen of GC, along with tislelizumab and radiation therapy. Following this intervention, the patient attained a clinical complete remission (CR). To date, there have been no documented instances of similar cases reaching complete remission using this treatment approach.

## Data Availability

The original contributions presented in the study are included in the article/supplementary material. Further inquiries can be directed to the corresponding author.
